# Fitness consultations in routine care of patients with type 2 diabetes in general practice: an 18-month non-randomised intervention study

**DOI:** 10.1186/1471-2296-11-83

**Published:** 2010-11-03

**Authors:** Henning Lohmann, Volkert Siersma, Niels F Olivarius

**Affiliations:** 1 General practice, Korsør, Denmark; 2 The Research Unit for General Practice and Section of General Practice, Department of Public Health, University of Copenhagen, Copenhagen, Denmark

## Abstract

**Background:**

Increasing physical activity is a cornerstone in the treatment of type 2 diabetes and in general practice it is a challenge to achieve long-term adherence to this life style change. The aim of this study was to investigate in a non-randomised design whether the introduction of motivational interviewing combined with fitness tests in the type 2 diabetes care programme was followed by a change in cardio-respiratory fitness expressed by VO_2max_, muscle strength of upper and lower extremities, haemoglobin A_1c _(HbA_1c_) and HDL-cholesterol.

**Methods:**

Uncontrolled 18-month intervention study with follow-up and effect assessment every 3 months in a primary care unit in Denmark with six general practitioners (GPs). Of 354 eligible patients with type 2 diabetes, 127 (35.9%) were included. Maximum work capacity was tested on a cycle ergometer and converted to VO_2max_. Muscle strength was measured with an arm curl test and a chair stand test. The results were used in a subsequent motivational interview conducted by one of the GPs. Patients were encouraged to engage in lifestyle exercise and simple home-based self-managed exercise programmes. Data were analysed with mixed models.

**Results:**

At end of study, 102 (80.3%) participants remained in the intervention. Over 18 months, VO_2max _increased 2.5% (p = 0.032) while increases of 33.2% (p < 0.001) and 34.1% (p < 0.001) were registered for the arm curl test and chair stand test, respectively. HDL-cholesterol increased 8.6% (p < 0.001), but HbA_1c _remained unchanged (p = 0.57) on a low level (6.8%). Patients without cardiovascular disease or pain from function limitation increased their VO_2max _by 5.2% (p < 0.0001) and 7.9% (p = 0.0008), respectively.

**Conclusions:**

In this 18-month study, participants who had repeated fitness consultations, including physical testing and motivational interviewing to improve physical activity, improved VO_2max_, muscle strength, and lipid profile. Our results indicate that physical testing combined with motivational interviewing is feasible in a primary health care setting. Here, a fitness consultation tailored to the individual patient, his/her comorbidities and conditions in the local area can be incorporated into the diabetes programme to improve patients' muscle strength and cardio-respiratory fitness.

## Background

Increases in cardio-respiratory fitness, muscle strength and level of physical activity are associated with decreased mortality and protect against age-related disabilities [1-7]. A substantial proportion of patients with type 2 diabetes have low levels of physical fitness and do not engage in the recommended level of physical activity [1,8]. These patients have increased cardiovascular mortality [9] and many comorbidities [10], e. g. hypertension, cardiovascular disease and arthritis, which may preclude some physical activities or require evaluation by a physician before the activities can be undertaken.

Regular exercise in type 2 diabetic individuals may have a significant effect on VO_2max _and may result in decreased HbA1c [11,12]. Similarly, HDL-cholesterol increases with cardio-respiratory fitness [13,14]. It has also been demonstrated that progressive resistance training increases muscle strength in type 2 diabetic patients [15].

Interventions that include fitness testing and individual exercise prescription are associated with more effect on fitness outcomes than interventions without these elements [16], and in recent years the use of individual behavioural approaches to increase physical activity has been advocated. It seems relevant to use motivational interviewing and include testing of muscle strength, cardio-respiratory fitness as well as exercise prescription in the motivational armamentarium of a diabetes care programme [17-22]. In a study of home-based resistance training in elderly people it was concluded that a positive attitude towards exercise and a sense of control over it were associated with adherence to the exercise regimen [23]. There are many laboratory studies with supervised exercise that demonstrate an effect on cardiovascular fitness and muscle strength, but results from general practice are scarce [11].

The over-all purpose of the present study was to see whether it was feasible for general practitioners (GPs) and their staff to motivate people with type 2 diabetes to increase and maintain their muscle strength and cardio-respiratory fitness by self-managed physical activities during an 18-month intervention period. We assumed that by providing patients with knowledge of their own muscle strength and cardio-respiratory fitness, they would become aware of discrepancies between their current physical fitness and personal goals for future health, and this realisation could induce behavioural changes [16,20]. In those patients who accepted to undergo the intervention, we measured a number of outcomes before, during and after the inclusion of fitness tests and motivational interviewing in the patients' regular diabetes control consultations. The primary outcomes were VO_2max _(maximal oxygen uptake, ml O_2 _kg^-1 ^min^-1^), muscle strength of upper and lower extremities, haemoglobin A_1c _(HbA_1c_), and HDL-cholesterol during the 18-month intervention period. Secondary outcomes were waist circumference, body mass index (BMI), systolic and diastolic blood pressure, fasting plasma glucose, total cholesterol, LDL-cholesterol, and triglycerides.

## Methods

### Study design and participants

The study was designed as an uncontrolled study in a primary health care unit with six GPs providing health services to approx. half of the 20,000 population of a town in Denmark. All health care providers in the unit participated. In Denmark, routine care of patients with type 2 diabetes is usually given by GPs and practice nurses in primary care units in a national structured diabetes programme recommending control every 3 months.

**Figure 1:**
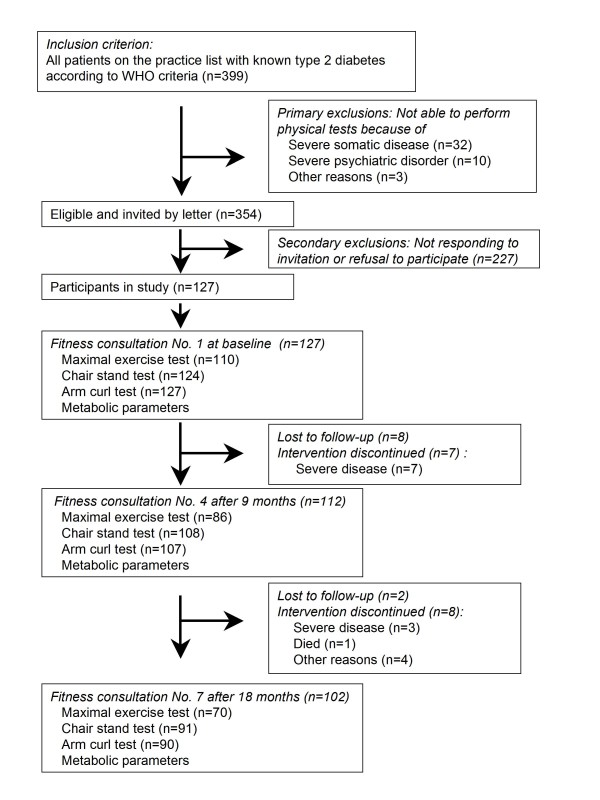
**Flow of patients through the trial and the number of different physical tests done at fitness consultation No. 1, 4 and 7**.

Of 399 patients with known type 2 diabetes, 127 were included (Figure 1). All were Caucasians. During the study, participants were considered lost to follow-up after non-response to one reminder. The reasons for exclusion or loss to follow-up appear from Figure 1. The ethics committee of West Zealand approved the protocol. Patients gave written informed consent.

### The fitness consultation

The six GPs involved were trained in the principles of aerobic and resistance exercise testing and training in a 3-hour session, and they were introduced to the strategies and techniques of motivational interviewing in another 3-hour session conducted by a researcher with wide experience of motivational interviewing.

During the 18 months of intervention, the patients were seen on two different days every three months in connection with the scheduled visits in the usual diabetes care programme.

On the first day, the practice nurse or the laboratory technician tested maximum work capacity (Watt_max_) and muscle strength and measured weight, height, waist circumference, blood pressure and HbA_1c_. At baseline and after 9 and 18 months, fasting plasma glucose, total cholesterol, HDL-cholesterol, fasting triglycerides, serum creatinine, HbA_1c_, and urinary albumin/creatinine-ratio were measured as well.

On the second day, the GPs, in conjunction with the usual diabetes control, carried out a fitness consultation. At every fitness consultation the GPs had to judge and note in the patient file the patient's position in relation to the Stages of change model (Pre-contemplation, Contemplation, Preparation, Action, Maintenance and Relapse). The consultation was then held bearing this knowledge in mind. The doctors and patients worked with ambivalent attitudes, using the results of the physical tests, the pro and cons of increased physical activity, resistance, readiness and ability to change. The GPs were recommended not to try to persuade patients to certain behaviour changes but to accept the choices made by the patients.

Results of the physical tests were presented to the patient using age- and sex-stratified nomograms and information about changes since the previous visit [2,24]. Life-style exercise, i.e. increasing physical activity in daily life, actions such as walking up and down stairs instead of using the lift, cycling instead of using the car, gardening etc., were suggested. A realistic goal of physical activity was negotiated, aiming at 2,500 kcal per week corresponding to approximately a half-hour walking and a half-hour cycling a day seven days a week. This is a level where the maximal effect on cardiovascular risk and HbA_1c _might be expected [4,25,26].

Self-managed resistance exercise was suggested for each of the major muscle groups three times a week gradually progressing to two or three sets with a resistance that could be done between a minimum of 10 times and a maximum of 15 times [24].

### Tests of fitness and muscle strength

Before testing, all patients had a 6-minute light warm-up period on the bicycle ergometer with 50% of maximum workload. Tests of muscle strength were done as described in "Senior Fitness Test" [2] by two tests: (a) an arm curl test during which the patient lifted weights from full extension to maximum flexion as many times as possible within 30 seconds. The outcome was the number of flexions; (b) a chair stand test where the patient stood up and sat down from a 40 cm high chair as many times as possible within 30 seconds. The outcome was the number of stand ups. The chair stand test is a measure of lower body strength and has a moderately high correlation to leg press scores (R = 0.78 for men and R = 0.71 for women). The arm curl test is a measure of upper body strength and has a moderately high correlation to combined 1-RM (repetition max) biceps, chest, and upper back (R = 0.84 for men and R = 0.79 for women). The test-retest reliability (95% confidence interval) for the chair stand test is 0.89 (0.79-0.93) and for the arm curl test 0.81 (0.72-0.88) [2].

Cardio-respiratory fitness was tested with an individualized symptom-limited ramp cycle ergometer (Monark^®^) test where we used a protocol with an individualized initial workload and a 12.5-watt increase per min. aiming at a testing time between six and 10 minutes. The results, i.e. the Watt_max _attained, were converted to VO_2max _(ml O_2 _kg^-1 ^min^-1^) by use of conversion formulas [27]. This method of measuring cardio-respiratory fitness has a high correlation, R = 0.97, with measurements of pulmonary ventilation and gas exchange [27].

We did not screen all participants with stress testing before exercise testing, but all were assessed clinically and with a resting ECG with regard to the risk of ischaemic heart disease and, at the discretion of the GP, referred to a cardiologist before testing [28]. Contraindications for the maximal exercise test were blood pressure > 180/110 mmHg, unstable angina pectoris, severe ischaemia on resting ECG, severe heart arrhythmia, aorta stenosis, pacemaker with fixed heart rate, autonomic neuropathy (resting heart rate > 100 bpm, orthostatic vertigo), proliferative retinopathy, and acute disease [24,28]. Patients on insulin treatment with blood glucose < 7 mmol/l were given 20 g glucose 15 minutes before testing. All tests were stopped if the patient felt unwell in any way. The health centre had access to resuscitation equipment including a heart starter.

### Biochemical and clinical variables

All blood samples were taken in the morning after an eight-hour overnight fast and a resting period of at least 15 min and no hard physical activity within the foregoing two hours. Samples were analysed at Slagelse Hospital. Fraction of HbA_1c _was measured by a high performance liquid chromatography method (a Tosoh Automated Glycohaemoglobin Analyzer HLC-723 G. Reference interval: 0.042-0.063). Serum total cholesterol concentration was measured enzymatically with cholesterol esterase-cholesterol oxidase-peroxidase reagent. Serum triglyceride concentrations were determined enzymatically with a lipase-glycerolkinase-glycerol-3-phosphate oxidase-peroxidase reagent. HDL-cholesterol was determined by a homogeneous enzymatic colorimetric method. Plasma glucose was measured by a hexokinase method. In freshly voided morning urine, creatinine was determined by a Jaffé reaction and albumin by an immunoturbidimetric method.

Body weight and height were measured without shoes and outer garments on the same scales throughout the study. BMI was calculated as (weight in kg)/(height in metres)^2^. Waist circumference was measured to the nearest cm in the mid-horizontal plane between lowest rib and iliac crest. Blood pressure was measured after 10 min. rest in the seated position as the lowest of three values using a mercury sphygmomanometer.

Pain with function limitation was defined as pain from joints and/or muscles in arms, shoulders, legs and/or back which reduced the performance at the physical tests at the 9- and/or 18-month follow-up as indicated by the patient. Cardiovascular disease (CVD) was defined as history of myocardial infarction and/or verified stenosis of coronary arteries and/or stroke and/or arteriosclerosis of the lower extremities verified by distal pressure measurement recorded at baseline and/or after 9 and/or 18 months.

### Statistical analysis

**Figure 2:**
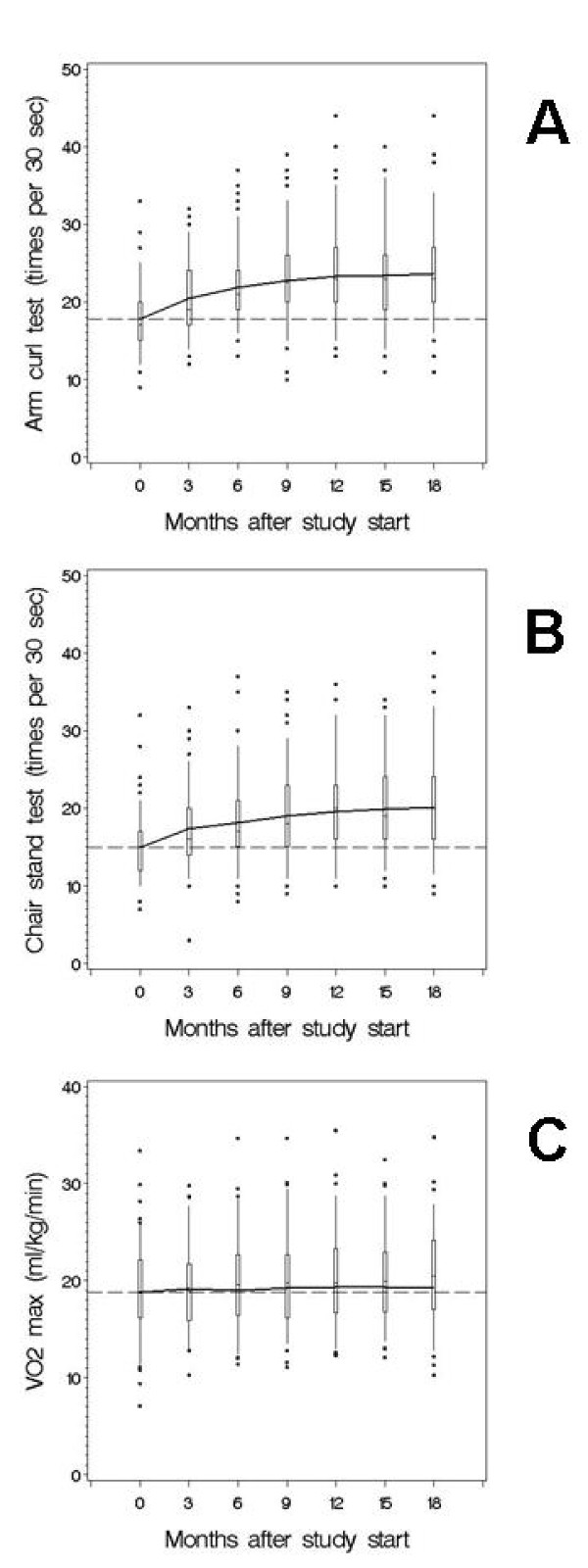
**Changes over 18 months in arm curl test (A), chair stand test (B), and VO_2max _(C)**.

Differences between study subjects and excluded patients were investigated by χ^2 ^tests or Kruskal-Wallis tests. The development of each outcome variable was described in a mixed model with a separate fixed effect for each examination, and a random patient intercept [29]. The concatenation of fixed effects was interpreted as the average development of the outcome, and was superimposed on the cross-sectional distributions of the outcome (shown as box-plots) in Figure 2. Whether the outcome remains the same over time was tested by a Wald test for the null hypothesis that all parameters of the fixed effects were the same. A heuristic measure of increase was Δ: the difference between the modelled baseline outcome and the modelled outcome at the seventh examination. A power calculation shows that the study has a power of 80% to detect a difference in change in VO_2max _from 0 (no change) to 1.3 ml O_2 _kg^-1 ^min^-1 ^during 18 months when n = 127.

Subgroup analyses were done by adding the stratification variable to the mixed model both as a main fixed effect and interacting with the fixed effects of the separate examinations. This allowed for completely different developments in the subgroups. The test for the significance of the difference between the developments was the Wald test for the null hypothesis that all parameters of fixed effects containing the subgroup variable were zero. Here Δ was the difference between the subgroup-specific modelled baseline and seventh examination outcome. Significance of the comparison tests was determined controlling for the false discovery rate at 5% [30]. Data were analysed with SAS PROC MIXED.

## Results

Of 354 eligible patients, 127 (35.9%) participated in the study. There was no statistically significant difference between the 227 non-responders and the 127 participants with regard to age (67.5/67.0 years, p = 0.77), gender (women, 42.3/42.5%, p = 0.96) and HbA_1c _(6.8/6.9%, p = 0.30) before the start of intervention (Figure 1). At baseline, participants were characterised by a low HbA_1c _and a high comorbidity (Table 1). Thirteen patients had atypical courses due to severe disease (myocardial infarction, stroke, accidents, and cancer) and one patient started participation in a placebo trial after inclusion. Test results from these patients were excluded from analysis from the date of the event.

**Table 1:** Baseline characteristics of the patients

Characteristic	
Sex (men/women)	73 (57%)/54 (43%)
Age (years)	67.0 (59.8-73.8)
Diabetes duration (years)	3 (2-8)
School education (basic/further)	39 (31%)/88 (69%)
Marital status (single/cohabiting)	29 (23%)/98 (77%)
VO_2max _(ml kg^-1 ^min^-1^)	19.0 (17.0-22.5)
Arm curl test (times per 30 sec)	17 (14-19)
Chair stand test (times per 30 sec)	14 (12-17)
Haemoglobin A_1c _(%)	6.8 (6.4-7.5)
HDL-cholesterol (mmol/l)	1.3 (1.1-1.5)
Total cholesterol (mmol/l)	5.0 (4.2-5.6)
LDL-cholesterol (mmol/l)	2.7 (2.2-3.3)
Fasting triglycerides (mmol/l)	1.5 (1.1-2.3)
Waist circumference (cm, men)	105 (101-115)
Waist circumference (cm, women)	99 (92-107)
Body mass index (kg/m^2^)	29.6 (26.3-33.3)
Systolic pressure (mmHg)	140 (130-150)
Diastolic pressure (mmHg)	80 (75-85)
Fasting plasma glucose (mmol/l)	7.7 (6.8-9.1)
Antihypertensive medication (N/Y)	36 (28%)/91 (72%)
Antidiabetic treatment (diet/oral agents/insulin)	37 (29%)/70 (55%)/20 (16%)
Statin treatment (N/Y)	49 (39%)/78 (61%)
Smoking (N/Y)	99 (78%)/28 (22%)
Albumin/creatinine ratio (≤ 3.5/> 3.5)	97 (76%)/30 (24%)
Cardiovascular disease^a ^(N/Y)	61 (48%)/66 (52%)
Pain with function limitation^b ^(N/Y)	53 (42%)/74 (58%)

^b ^Recorded after 9 and/or 18 months

### Primary outcome measures

Over the 18 months, clear average increases of approximately one third were obtained for both arm curl test and chair stand test, while VO_2max _increased moderately (Table 2). The average increase in muscle strength abated over the 18 months but never declined (Figure 2). HDL-cholesterol also increased, but HbA_1c _remained unchanged on a low level.

**Table 2:** Developments in primary outcome measures during the study according to baseline variables

			VO_2max_ (ml/kg/min)				Arm curl test (times per 30 sec)				Chair stand test (times per 30 sec)				Haemoglobin A_1c_ (%)				HDL-cholesterol (mmol/l)			
			
		*n*	Δ	SE	%	*p *^a^	Δ	SE	%	*p *^a^	Δ	SE	%	*p *^a^	Δ	SE	%	*p *^a^	Δ	SE	%	*p *^a^
Total		127	0.46	0.20	2.5	0.032	5.9	0.30	33.2	< 0.0001	5.1	0.31	34.1	< 0.0001	0.020	0.066	0.3	0.57	0.11	0.02	8.6	< 0.0001
Sex	Male	73	0.58	0.26	2.9	*	6.3	0.40	34.2		5.6	0.40	36.4		-0.069	0.088	-1.0		0.09	0.03	7.0	
	Female	54	0.34	0.31	2.0		5.3	0.46	31.8		4.4	0.47	31.0		0.129	0.098	1.8		0.14	0.03	10.2	
Age	≤ 67 years	63	0.58	0.26	2.7	*	7.0	0.42	37.9	*	6.0	0.41	37.9	*	0.042	0.092	0.6		0.10	0.03	8.1	
	> 67 years	64	0.32	0.31	2.0		4.7	0.43	27.8		4.1	0.45	29.2		-0.002	0.096	-0.0		0.13	0.03	9.3	
Diabetes duration	≤ 1 year	31	0.16	0.37	0.8		7.0	0.60	38.4		6.8	0.58	45.2	*	-0.066	0.130	-1.0		0.14	0.04	10.0	
	> 1 year	96	0.58	0.23	3.2		5.5	0.35	31.2		4.5	0.36	30.0		0.049	0.077	0.7		0.11	0.03	8.1	
School education	Further	88	0.56	0.23	2.9		6.1	0.36	33.3		4.9	0.36	31.4		0.079	0.078	1.1		0.08	0.03	6.2	
	Basic	39	0.21	0.38	1.2		5.3	0.57	32.6		5.7	0.58	42.0		-0.128	0.123	-1.8		0.19	0.04	14.8	
Marital status	Single	29	-0.37	0.49	-2.3		3.9	0.68	23.0		3.4	0.71	24.2	*	0.168	0.153	2.3		0.14	0.05	10.7	
	Co-habiting	98	0.62	0.22	3.2		6.4	0.34	35.4		5.5	0.34	36.4		-0.013	0.073	-0.2		0.11	0.02	8.2	
BMI	< 29.62 kg/m^2^	64	0.48	0.28	2.5		5.9	0.43	31.9		5.6	0.43	36.7		0.009	0.094	0.1		0.14	0.03	10.2	*
	≥ 29.62 kg/m^2^	63	0.44	0.29	2.4		5.9	0.43	34.7		4.5	0.44	31.2		0.030	0.093	0.4		0.09	0.03	6.9	
Waist circum-ference	< ♂/♀ median	60	0.84	0.27	4.3		5.9	0.44	32.0		5.6	0.43	36.6		-0.001	0.096	-0.0		0.10	0.03	7.4	
	≥ ♂/♀ median	67	0.04	0.29	0.2		5.9	0.43	34.3		4.6	0.44	31.4		0.039	0.092	0.5		0.12	0.03	9.8	
Systolic pressure	≤ 130 mmHg	50	0.48	0.32	2.6		5.6	0.50	31.2		3.9	0.50	25.9		0.137	0.109	1.9		0.11	0.04	8.5	
	> 130 mmHg	75	0.51	0.26	2.7		6.3	0.39	35.5		6.0	0.40	40.0		-0.050	0.086	-0.7		0.12	0.03	8.8	
Diastolic pressure	≤ 80 mmHg	89	0.27	0.25	1.5		5.9	0.37	32.9		5.0	0.38	33.4		0.063	0.081	0.9		0.12	0.03	9.0	
	> 80 mmHg	36	0.93	0.35	4.9		6.2	0.54	36.3		5.6	0.55	36.9		-0.070	0.121	-1.0		0.10	0.04	8.2	
Antihypertensive medi-cation	No	36	1.07	0.35	5.4		7.3	0.57	40.6		6.1	0.58	40.1		0.128	0.127	1.9		0.16	0.04	12.1	
	Yes	91	0.17	0.24	0.9		5.3	0.36	30.2		4.7	0.36	31.7		-0.019	0.077	-0.3		0.10	0.03	7.3	
Albumin/ creatinine ratio	≤ 3.5	97	0.58	0.22	3.1		6.7	0.34	38.1	*	5.8	0.35	39.1	*	0.033	0.075	0.5	*	0.12	0.02	8.7	
	> 3.5	30	-0.05	0.45	-0.3		3.4	0.60	18.5		2.9	0.61	19.5		-0.036	0.134	-0.5		0.10	0.04	8.2	
Fasting plasma glucose	≤ 7 mmol/l	38	0.21	0.36	1.1		6.6	0.54	37.0		6.1	0.55	42.3		0.064	0.119	1.0	*	0.11	0.04	7.9	
	> 7 mmol/l	89	0.58	0.24	3.2		5.6	0.37	31.5		4.6	0.37	30.5		-0.005	0.079	-0.1		0.11	0.03	8.9	
Diabetes treatment	Diet alone	37	0.42	0.36	2.2		7.0	0.57	38.8		6.7	0.55	44.8	*	0.128	0.126	2.0		0.16	0.04	11.3	
	Oral agents	70	0.73	0.27	3.9		5.9	0.40	33.7		5.0	0.41	32.9		-0.016	0.089	-0.2		0.11	0.03	8.5	
	Insulin	20	-0.31	0.54	-1.7		3.9	0.75	21.8		2.4	0.78	16.9		-0.041	0.159	-0.6		0.06	0.05	4.5	
Statin treatment^b^	None	38	1.36	0.36	7.5		6.3	0.56	36.1		5.3	0.57	37.8		0.192	0.120	2.7		0.18	0.04	13.3	
	Un-changed	78	0.16	0.26	0.9		5.9	0.39	33.0		5.0	0.40	33.0		-0.061	0.086	-0.9		0.08	0.03	5.8	
	Initiated	11	-0.33	0.62	-1.7		4.8	0.92	26.5		5.0	0.91	31.2		-0.043	0.204	-0.6		0.13	0.07	9.7	
Smoking	No	99	0.45	0.21	2.3		5.9	0.33	33.2		5.2	0.34	33.9		0.115	0.072	1.6	*	0.09	0.02	6.7	
	Yes	28	0.55	0.54	3.1		5.6	0.72	32.6		4.9	0.77	35.0		-0.454	0.157	-6.3		0.24	0.05	18.5	
Pain with function limitation	No	53	1.53	0.32	7.9	*	6.0	0.49	32.4		5.7	0.48	37.7		-0.006	0.108	-0.1		0.17	0.03	12.2	
	Yes	74	-0.15	0.25	-0.8		5.9	0.39	34.1		4.7	0.40	32.0		0.036	0.084	0.5		0.08	0.03	6.5	
Cardiovascular disease	No	66	1.03	0.23	5.2	*	6.0	0.39	33.3		5.5	0.38	35.2		0.012	0.084	0.2		0.12	0.02	8.9	
	Yes	45	-0.77	0.37	-4.3		5.7	0.54	32.6		4.1	0.55	29.4		-0.082	0.118	-1.1		0.05	0.03	3.8	

*n *is number of patients. Δ is a measure of absolute increase defined as the difference between the (subgroup-specific) modelled baseline and the 18-month examination outcome. The corresponding increase, relative to the modelled baseline examination outcome, is indicated under %. ^a ^Differences between subgroups are assessed by a Wald test for the time-subgroup interaction. To account for multiple testing, the tests that remained significant after controlling for the false discovery rate at 5% are indicated with a *. ^b ^Indicates whether patients had unchanged statin treatment during the study or stated statin treatment during the study.

### Subgroup analyses

The development of VO_2max _varied with age, sex, CVD and pain with function limitation (Table 2). In a full multivariate model including all the baseline variables listed in Table 2 as predictors and VO_2max _as outcome, only CVD (p = 0.001) and pain with function limitation (p = 0.023) were statistically significant. Patients without cardiovascular disease or pain from function limitation increased their VO_2max _by 5.2% (p < 0.0001) and 7.9% (p = 0.0008), respectively. All subgroups increased their muscle strength, but high age and microalbuminuria were associated with relatively small improvements in muscle strength tests.

### Secondary outcome measures

Waist circumference, BMI and fasting plasma glucose did not change, and there was a slight increase in systolic and diastolic pressure. The lipid profile improved (Table 3).

**Table 3:** Changes in secondary outcome measures during the study period

Secondary outcome measure	Δ	SE	*p*
Waist circumference (cm)	0.28	0.54	0.93
BMI (kg/m^2^)	0.13	0.09	0.13
Systolic blood pressure (mmHg)	2.50	1.83	0.037
Diastolic blood pressure (mmHg)	1.38	0.92	0.0009
Fasting plasma glucose (mmol/l)	0.12	0.19	0.79
Total cholesterol (mmol/l)	-0.25	0.08	0.006
LDL-cholesterol (mmol/l)	-0.31	0.07	< 0.0001
Triglycerides (mmol/l)	-0.09	0.14	0.66

Δ is defined as the difference between the modelled baseline and the 18-month examination outcome. SE, standard error. n = 127. For baseline values see Table 1

### The course of the intervention

Ten of the 127 participants were referred to initial supervision by a physiotherapist, and two attended local fitness centres, but most chose lifestyle exercise or self-managed home-based exercise programmes or both. The programmes included aerobic training using an exercise bike and resistance training with weights or use of own body weight [2]. The intervention was safe and well tolerated with a dropout rate of 19.6% despite a high degree of comorbidity (Table 1). One maximal exercise test was stopped because the patient felt unwell, but there were no other complications associated with the test procedures except for slight tenderness of joints and muscles. Reasons for not performing all three tests at the final session were musculoskeletal disease (14/102), blood pressure >180/110 (7/102), heart disease (9/102), and acute illness (1/102). The extra time attributed to the expansion of the usual diabetes control to include a fitness consultation was estimated to be 10 min.

## Discussion

In this 18-month uncontrolled intervention study, repeated fitness consultations including fitness testing and motivational interviewing resulted in the participants having increased muscle strength and VO_2max_, and an improved lipid profile, while HbA_1c _remained unchanged on a low level. Among the secondary outcome measures, waist circumference, BMI and fasting plasma glucose were unchanged, blood pressures increased slightly, whereas total cholesterol and LDL-cholesterol decreased.

### Comparison with relevant literature

In the studies by David et al. [31,32], six months of supervised progressive high-intensity resistance training three times a week in older (mean age 67.6 years) type 2 diabetic patients with few comorbidities resulted in a 41.7% increase in upper body muscle strength and a 28.0% increase in lower body muscle strength. Additional home-based resistance training for 6 months was effective in maintaining the gymnasium-based improvements in muscle strength. These results are comparable with the increase and maintenance of muscle strength attained in the present study considering its less intensive intervention (Table 2 and Figure 2).

A meta-analysis has reported an 11.8% increase of VO_2max _in structured aerobic exercise studies with the following average characteristics: 3.4 sessions per week, 49 min. per session for 20 weeks with exercise intensities of 50-75% of VO_2max _[11]. The study populations in the meta-analysis were selected so they had a minimum of cardiovascular or orthopaedic limitations and were on average 12 years younger than the present study population. During our intervention, there was a slight (2.4%) but significant increase in VO_2max _for the whole group. There was a more substantial increase of 7,9% in the subgroup (n = 53) without function-limiting pain and 5,2% in the subgroup (n = 61) without CVD (Table 2). In light of these post hoc explanatory subgroup analyses, our study indicated that physical tests and motivational interviewing had an impact comparable with supervised exercise sessions on muscle strength and VO_2max_.

There is a steep inverse relationship between cardio-respiratory fitness and mortality in men with documented diabetes [5]. This could mean that an improvement in fitness, like the one we observed, is of clinical significance. In an observational prospective study of men, an increase of 7.0 ml/kg/min in VO_2max _over 4.9 years was associated with an estimated reduction of 30% in mortality risk during the following 5.1 years [7]. HbA_1c _did not change in participants during the present study, which perhaps is explained by the low baseline level of HbA_1c _[33]. The increase in HDL-cholesterol may be a result of increased muscle strength and increased VO_2max _[34]. The intervention had no impact on BMI and waist circumference. Nevertheless the intervention may have had a clinically significant effect on health as the inverse gradient between fitness and mortality in men with documented diabetes mentioned above is independent of BMI [4,5]. The improvement in glycaemic control following endurance and strength training may also be observed with unchanged BMI [35].

### Strengths and limitations of the study

The study was done in the setting of a primary health care unit using primary care practitioners to carry out the intervention. The whole town was aware of the project, which made it difficult and demanding to do a randomised controlled trial, e.g. using the idea of waiting list controls. Unlike most other studies, patients with cardiovascular or musculoskeletal disease were not excluded [11]. The idea of physical testing was completely new to the patients and this, in combination with the relatively high median age, was probably the major reason for the low participation rate.

The lack of a control group is a major limitation of the study which leaves the possibility that the improvements in outcomes could be due to the general development in the natural history of type 2 diabetes and, for the physical tests in particular, to some degree of habituation. However, it is unlikely that the observed improvements in cardio-respiratory fitness and muscle strength can be explained entirely by these effects. Firstly, control groups in previous randomised studies show a decrease in VO_2max _of 1% over a period of 20 weeks and small non-significant increases of 1.5% and 5% in upper and lower body strength [11,31]. Secondly, the expected age-related decline in muscle strength and VO_2max _over 18 months can be estimated to be 2.5% and 2.2% respectively in the population of the present study [2,36]. Thirdly, if habituation explained the improvement, the same development should be expected in the different subgroups, and this is not the case (Table 2).

The arm curl test and the chair stand test are validated methods for measuring muscle strength in upper and lower extremities. These tests have a high test-retest reliability, are simple to use in everyday practice, and can be done by nearly all patients [2] (Figure 1). Until now, experience with these tests has been limited to a population over 60 years of age. In this study, it was assumed that they could also be used with younger people to measure changes in muscle strength. The bicycle ergometer test is known to give an accurate estimate of VO_2max _[24,27], but about one third of the type 2 diabetic participants were unable to do the test because of contraindications and comorbidity (Figure 1). However, all the participants were able to perform at least one of the three tests so all got a result that could be used in the motivational interview.

## Conclusion

Clinical implementation of increased physical activity in the treatment of type 2 diabetes is still far from being standard practice. Our results indicate that physical testing combined with motivational interviewing can be done in a primary health care setting. Here, a fitness consultation tailored to the individual patient, his/her comorbidities and conditions in the local area can be incorporated into the diabetes programme to improve patients' muscle strength and cardio-respiratory fitness. The extra workload caused by fitness consultations in primary care could for instance be carried by practice nurses or physiotherapists who have received training in motivational interviewing and physical testing. Randomised trials are needed to confirm our findings and to optimise recommendations for the content and the frequency of the fitness consultations [37].

## Competing interests

The authors declare that they have no competing interests.

## Authors' contributions

HL made substantial contributions to conception and design of the study, planned and conducted it, collected, analysed and interpreted data and drafted the manuscript,. VS did the statistical analysis and interpreted data and has been involved in drafting the manuscript and revising it critically for important intellectual content.,. NO made substantial contributions to conception, design, analysis and interpretation. He has revised the manuscript critically for important intellectual content.

All authors read and approved the final manuscript.

## Funding

The study was supported by The Danish Board of Health and the Region of West-Zealand.

## Pre-publication history

The pre-publication history for this paper can be accessed here:

http://www.biomedcentral.com/1471-2296/11/83/prepub

## References

[B1] Wei M Gibbons LW Kampert JB Nichaman MZ Blair SN Low cardiorespiratory fitness and physical inactivity as predictors of mortality in men with type 2 diabetes Ann Intern Med 2000 132 605 611 10766678 10.7326/0003-4819-132-8-200004180-00002

[B2] Rikli RE Jones CJ Senior Fitness Test Manual 2001 Human Kinetics, Champaign IL

[B3] Rantanen T Muscle strength, disability and mortality Scand J Med Sci Sports 2003 13 3 8 10.1034/j.1600-0838.2003.00298.x 12535311

[B4] Hu G Jousilahti P Barengo NC Qiao Q Lakka TA Tuomilehto J Physical activity, cardiovascular risk factors, and mortality among Finnish adults with diabetes Diabetes Care 2005 28 799 805 10.2337/diacare.28.4.799 15793176

[B5] Church TS Cheng YJ Earnest CP Barlow CE Gibbons LW Priest EL Blair SN Exercise capacity and body composition as predictors of mortality among men with diabetes Diabetes Care 2004 27 83 88 10.2337/diacare.27.1.83 14693971

[B6] Blair SN Kohl HW III Paffenbarger RS Jr Clark DG Cooper KH Gibbons LW Physical fitness and all-cause mortality. A prospective study of healthy men and women Jama 1989 262 2395 2401 10.1001/jama.262.17.2395 2795824

[B7] Blair SN Kohl HW III Barlow CE Paffenbarger RS Jr Gibbons LW Macera CA Changes in physical fitness and all-cause mortality. A prospective study of healthy and unhealthy men Jama 1995 273 1093 1098 10.1001/jama.273.14.1093 7707596

[B8] Plotnikoff RC Taylor LM Wilson PM Courneya KS Sigal RJ Birkett N Raine K Svenson LW Factors associated with physical activity in Canadian adults with diabetes Med Sci Sports Exerc 2006 38 1526 1534 10.1249/01.mss.0000228937.86539.95 16888470

[B9] Gu K Cowie CC Harris MI Mortality in adults with and without diabetes in a national cohort of the U.S. population, 1971-1993 Diabetes Care 1998 21 1138 1145 10.2337/diacare.21.7.1138 9653609

[B10] Struijs JN Baan CA Schellevis FG Westert GP van den Bos GA Comorbidity in patients with diabetes mellitus: impact on medical health care utilization BMC Health Serv Res 2006 6 84 10.1186/1472-6963-6-84 16820048 PMC1534031

[B11] Boule NG Kenny GP Haddad E Wells GA Sigal RJ Meta-analysis of the effect of structured exercise training on cardiorespiratory fitness in Type 2 diabetes mellitus Diabetologia 2003 46 1071 1081 10.1007/s00125-003-1160-2 12856082

[B12] Boule NG Weisnagel SJ Lakka TA Tremblay A Bergman RN Rankinen T Leon AS Skinner JS Wilmore JH Rao DC Effects of exercise training on glucose homeostasis: the HERITAGE Family Study Diabetes Care 2005 28 108 114 10.2337/diacare.28.1.108 15616242

[B13] Boule NG Bouchard C Tremblay A Physical fitness and the metabolic syndrome in adults from the Quebec Family Study Can J Appl Physiol 2005 30 140 156 15981784 10.1139/h05-111

[B14] Verity LS Ismail AH Effects of exercise on cardiovascular disease risk in women with NIDDM Diabetes Res Clin Pract 1989 6 27 35 10.1016/0168-8227(89)90054-5 2649339

[B15] Ibanez J Izquierdo M Arguelles I Forga L Larrion JL Garcia-Unciti M Idoate F Gorostiaga EM Twice-weekly progressive resistance training decreases abdominal fat and improves insulin sensitivity in older men with type 2 diabetes Diabetes Care 2005 28 662 667 10.2337/diacare.28.3.662 15735205

[B16] Nielsen PJ Hafdahl AR Conn VS Lemaster JW Brown SA Meta-analysis of the effect of exercise interventions on fitness outcomes among adults with type 1 and type 2 diabetes Diabetes Res Clin Pract 2006 74 111 120 10.1016/j.diabres.2006.03.033 16735074

[B17] Rubak S Sandbaek A Lauritzen T Christensen B Motivational interviewing: a systematic review and meta-analysis Br J Gen Pract 2005 55 305 312 15826439 PMC1463134

[B18] Kirk AF Mutrie N MacIntyre PD Fisher MB Promoting and maintaining physical activity in people with type 2 diabetes Am J Prev Med 2004 27 289 296 10.1016/j.amepre.2004.07.009 15488358

[B19] Rollnick S Mason P Butler C Health Behavior Change: A guide for practitioners 1999 London: Churchill Livingstone

[B20] Britt E Hudson SM Blampied NM Motivational interviewing in health settings: a review Patient Educ Couns 2004 53 147 155 10.1016/S0738-3991(03)00141-1 15140454

[B21] Di Loreto C Fanelli C Lucidi P Murdolo G De CA Parlanti N Santeusanio F Brunetti P De FP Validation of a counseling strategy to promote the adoption and the maintenance of physical activity by type 2 diabetic subjects Diabetes Care 2003 26 404 408 10.2337/diacare.26.2.404 12547870

[B22] Kirk AF Barnett J Mutrie N Physical activity consultation for people with Type 2 diabetes Evidence and guidelines. Diabet Med 2007 24 809 816 10.1111/j.1464-5491.2007.02190.x 17650156

[B23] Jette AM Rooks D Lachman M Lin TH Levenson C Heislein D Giorgetti MM Harris BA Home-based resistance training: predictors of participation and adherence Gerontologist 1998 38 412 421 9726128 10.1093/geront/38.4.412

[B24] American college of sports medicine ACSM´s Guidelines for Exercise Testing and Presciption 2000 Lippincott Williams & Wilkins, Philadelphia

[B25] Hu FB Stampfer MJ Solomon C Liu S Colditz GA Speizer FE Willett WC Manson JE Physical activity and risk for cardiovascular events in diabetic women Ann Intern Med 2001 134 96 105 11177312 10.7326/0003-4819-134-2-200101160-00009

[B26] Di Loreto C Make Your Diabetic Patients Walk Diabetes Care 2005 28 1295 1302 10.2337/diacare.28.6.1295 15920042

[B27] Storer TW Davis JA Caiozzo VJ Accurate prediction of VO2max in cycle ergometry Med Sci Sports Exerc 1990 22 704 712 10.1249/00005768-199010000-00024 2233211

[B28] Sigal RJ Kenny GP Wasserman DH Castaneda-Sceppa C White RD Physical activity/exercise and type 2 diabetes: a consensus statement from the American Diabetes Association Diabetes Care 2006 29 1433 1438 10.2337/dc06-9910 16732040

[B29] Verbeke G Molenberghs G Linear Mixed Models for Longitudinal Data 2000 Springer series in statistics. Springer, New York

[B30] Benjamini Y H Y "Controlling for the false discovery rate: a practical and powerful approach to multiple testing" Journal of the Royal Statistical Society 1995 57 1 289 300

[B31] Dunstan DW Daly RM Owen N Jolley D De Court Shaw J Zimmet P High-intensity resistance training improves glycemic control in older patients with type 2 diabetes Diabetes Care 2002 25 1729 1736 10.2337/diacare.25.10.1729 12351469

[B32] Dunstan DW Daly RM Owen N Jolley D Vulikh E Shaw J Zimmet P Home-based resistance training is not sufficient to maintain improved glycemic control following supervised training in older individuals with type 2 diabetes Diabetes Care 2005 28 3 9 10.2337/diacare.28.1.3 15616225

[B33] Sigal RJ Kenny GP Boule NG Wells GA Prud'homme D Fortier M Reid RD Tulloch H Coyle D Phillips P Effects of aerobic training, resistance training, or both on glycemic control in type 2 diabetes: a randomized trial Ann Intern Med 2007 147 357 369 17876019 10.7326/0003-4819-147-6-200709180-00005

[B34] Balducci S Leonetti F Di MU Fallucca F Is a long-term aerobic plus resistance training program feasible for and effective on metabolic profiles in type 2 diabetic patients? Diabetes Care 2004 27 841 842 10.2337/diacare.27.3.841 14988317

[B35] Cauza E Hanusch-Enserer U Strasser B Ludvik B Metz-Schimmerl S Pacini G Wagner O Georg P Prager R Kostner K The relative benefits of endurance and strength training on the metabolic factors and muscle function of people with type 2 diabetes mellitus Arch Phys Med Rehabil 2005 86 1527 1533 10.1016/j.apmr.2005.01.007 16084803

[B36] Paterson DH Cunningham DA Koval JJ St Croix CM Aerobic fitness in a population of independently living men and women aged 55-86 years Med Sci Sports Exerc 1999 31 1813 1820 10.1097/00005768-199912000-00018 10613433

[B37] Praet SF van Loon LJ Exercise: the brittle cornerstone of type 2 diabetes treatment Diabetologia 2008 51 398 401 10.1007/s00125-007-0910-y 18183362 PMC2668613

